# Effectiveness of post-campaign, door-to-door, hang-up, and communication interventions to increase long-lasting, insecticidal bed net utilization in Togo (2011–2012): a cluster randomized, control trial

**DOI:** 10.1186/1475-2875-13-260

**Published:** 2014-07-09

**Authors:** Rachelle E Desrochers, Kendra Siekmans, Peter R Berti, Karen Bramhill, Sarah AW Buchan, Guy K Battah, Dodji Gbetoglo, Kokou Vignikin, Alice Sabino

**Affiliations:** 1 HealthBridge, 1004-1 Nicholas Street, Ottawa, ON K1N 7B7, Canada; 2 International Federation of Red Cross and Red Crescent Societies, PO Box 303, CH-1211 Geneva 19, Switzerland; 3 Togolese Red Cross, PO Box 655, 51 Sahoudè Street, Amoutivé, Lomé, Togo; 4 Unité de recherche démographique, Université de Lomé, PO Box 12971, Lomé, Togo; 5 Independent Consultant for the International Federation of Red Cross and Red Crescent Societies, London, UK

**Keywords:** Hang-up, Behaviour-change communication, Insecticide-treated net use, Mass long-lasting insecticide net distribution campaign, Community health worker, Universal coverage, Togo

## Abstract

**Background:**

It is well established that insecticide-treated bed nets (ITNs), in particular long-lasting, insecticidal nets (LLINs), can be used as one of the primary interventions for effective malaria control. A consistent gap between net ownership and use has been observed, indicating that factors exist that prevent an owned mosquito net from being used. One approach used in the context of LLIN campaigns is a post-distribution, door-to-door visit of households with educational messages and to physically assist with hang-up of nets.

**Methods:**

A cluster randomized trial was conducted in the Plateaux Region of Togo to evaluate the effectiveness of different approaches to post-LLIN campaign home visits (number of visits and timing) by volunteers to enhance LLIN hang-up and utilization.

**Results:**

It was found that, in general, households that received intervention visits, particularly the most recent intervention visit, had levels of use that were typically 5 to 10% higher than the control households, while access did not differ among control and intervention households. Eight months post-campaign, ITN use by all individuals, children under five years and women of reproductive age was 11.3 to 14.4 percentage points greater in the study arm that received all three intervention visits than in the control communities. In households that received one or two additional door-to-door visits, the majority of respondents indicated that the volunteer provided new information during the visit regarding the use and importance of ITNs despite having received previous multiple visits.

**Conclusions:**

The impact of the interventions appears to have been primarily through the delivery and reinforcement of key behaviour-**c**hange **c**ommunication (BCC) messages regarding the importance of using an ITN and its care. Regardless of whether the respondents in fact received new information or had forgotten earlier information, this suggests that regular visits from community agents are useful in reinforcing key BCC messages.

## Background

Insecticide-treated bed nets (ITNs), in particular long-lasting, insecticidal nets (LLINs), are a key intervention for malaria control. If full coverage were achieved, ITNs could reduce child mortality by an average of 17% compared to no nets, in sub-Saharan Africa [1], so that at least five lives would be saved per year for every 1,000 children aged under five years using an ITN [2]. Mass ITN distribution campaigns are frequently adopted to achieve rapid scale-up in household ITN coverage [3].

A gap between levels of ITN ownership (generally defined as ownership of at least one ITN at the household-level) and levels of use has been consistently observed, where use is considerably lower than ownership [4]. The gap between ownership and ITN use in the population that has access to an ITN within their household (assuming that one ITN covers two people) is much smaller but still remains [5]. The reasons for non-use of owned and available ITNs are likely complex and, although recently significant progress has been made in understanding these factors [6,7], many questions remain unanswered. To address the access – use gap, one approach is the door-to-door visit of households with educational messages and assisting ITN recipients with hang-up of nets [8]. These so-called ‘hang-up’ visits are often carried out by community agents following mass campaigns or prior to peak transmission periods to ensure ITNs are hanging and used.

The effect of door-to-door visits on ITN hanging and utilization appears to depend on the baseline rate of ITN utilization (net culture), the effect of seasonality and on whether the reasons for non-use are adequately addressed in the behaviour-change communication (BCC) messages provided. In a review undertaken in 2008, three studies were identified that explored the impact of such hang-up activities and found increases in ITN use varying from 3 to 8% in countries where use was high (i e, 70% or higher) [7]. After the review was published, another survey in Togo showed a difference of 24 percentage points in the proportion of ITNs hanging eight months after a campaign when initial hanging rates were moderate (54%) [9].

The organization of door-to-door visits is costly, even when undertaken by community volunteers, therefore more study is needed to assess whether this approach should be recommended for use in all campaigns or in which situations it can be expected to be beneficial. To this end, a cluster randomized control trial carried out in the Plateaux Region of Togo. In October 2011, the Ministry of Health (MoH) and its partners conducted a universal coverage mass distribution campaign of LLINs throughout the country. The primary objective of the study was to evaluate the effectiveness of varying number and timing of hang-up visits on LLIN utilization in a nine-month period following the mass distribution campaign. The hypothesis was that, in this setting with low or moderate net use (less than 50 or 70% of existing nets are being used, respectively), one or more hang-up visits would increase ITN utilization by at least 15% over the course of the study, irrespective of other effects such as general messages during the campaign or season.

## Methods

### Setting

The study took place in four of the 12 districts (Wawa, Amou, Danyi and Akebou) of the Plateaux Region in Togo with a total population of 286,567 [10]. Malaria transmission in the Plateaux Region of Togo reflects the rainfall patterns, with a single, long, stable, rainy reason (March to October) [11]. This area was selected due to the high prevalence of malaria and the alignment between the research objectives and the National Malaria Control Programme’s strategy. Malaria is a major public health problem in Togo, accounting for 49% of health consultations and 42% of hospitalizations in 2010 [12].

In Togo, the use of ITNs is promoted for the prevention of malaria through routine health services and periodic campaign distributions. The distribution of ITNs by campaigns has been conducted previously in 2004 and 2008, with free ITNs given to children under five years of age as part of national integrated child health campaigns. In September 2011, the MoH and its supporting partners conducted a universal coverage LLIN distribution campaign, integrated with other health interventions. For the 2011 campaign, the policy for reaching universal coverage in beneficiary households was based on one ITN for every two persons. However, if the household already had nets that were in good condition, these existing nets were taken into account when calculating the number of new nets a household was eligible to receive.

### Description of interventions

**Figure 1:**
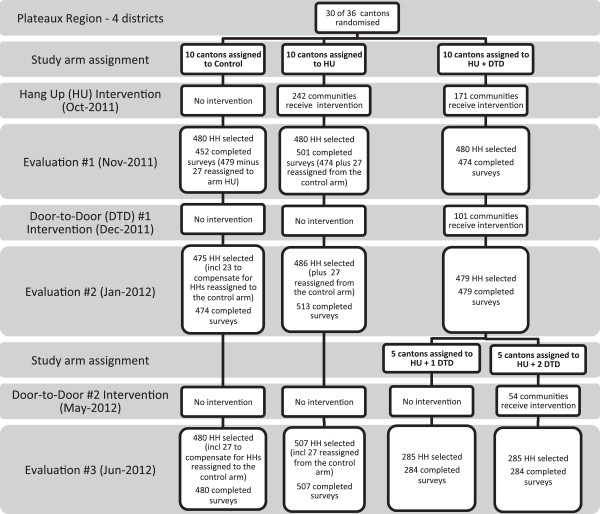
Overview of the study design depicting the flow of clusters and households through each phase of the study.

A cluster randomized design was used to minimize contamination between study arms by allocating groups of communities (cantons) to each study arm [13]. Moreover, there may be population-level effects of the intervention when applied to a large proportion of a population simultaneously [14]. See Figure 1 for an overview of the study design.

The research study consisted of three intervention periods, each followed by a household survey approximately one month post-intervention to evaluate ITN use in intervention and control areas using restricted randomization. For the purposes of this study, a cluster is defined as a canton. Thirty of the 36 cantons in the four districts were selected and assigned to one of three study arms. Restricted randomization was used to ensure an acceptable balance among study arms, including similar proportion of cantons assigned from each district, distribution of these cantons across study arms and similar average cluster population size across study arms. More specifically, the balance criteria used were: approximately 80% of the cantons in each prefecture (region) were to be included in the study; a minimum of 1 (in the smallest prefecture) or 2 (in the three larger prefectures) cantons were to be assigned to each study arm; and a minimum of 2 large (>10,000), 2 medium (5,000-10,000) and 2 small (<5,000) cantons were to be assigned to each arm (based on estimated canton population from 2010 census data).

The control arm consisted of ten cantons that received no intervention until after the study was completed, representing baseline use over the course of the study. The remaining 20 cantons received the first intervention, a post-campaign hang-up visit. Ten of these were assigned to a hang-up visit only arm (HU) and did not receive any further interventions. The other ten cantons were assigned to a hang-up visit and additional door-to-door visit arm (HU + DTD). These ten cantons received a dry season door-to-door visit. After the first door-to-door, the decision was made to split the HU + DTD arm into two groups of five cantons each, where only one of the two groups received the third intervention, a rainy season door-to-door visit. The five cantons that received the hang-up visit and only the first door-to-door visit will be referred to as HU + 1 DTD while the five cantons that received both door-to-door visits will be referred to as HU + 2 DTD.

The first intervention, the hang-up visits, occurred immediately following completion of the national campaign for distribution of the LLINs to households toward the end of the rainy season. In October 2011, Togo Red Cross (TRC) volunteers conducted house-to-house visits in the 20 cantons of arms HU, HU + 1 DTD and HU + 2 DTD. Volunteers visited approximately 15 households per day. During each visit, the volunteers, supplied with string and nails, physically assisting households with the hanging the ITNs and delivered standardized messages on malaria, the importance of using an ITN, and corrections of misconceptions regarding malaria and ITNs. The impact of this first intervention was evaluated one month later (November 2011). The second intervention occurred only a few weeks after the first evaluation (December 2011) to coincide with the height of the dry season. TRC volunteers conducted door-to-door visits in ten of the 20 cantons that received the first hang-up visit (HU + DTD) to reinforce the same key standardized messages regarding the importance of using an ITN and its care. Physical assistance with the hanging of ITNs was not an explicit part of the second phase of the intervention but volunteers did assist with hanging when requested to so. The second evaluation took place in mid-January 2012. The third and final intervention was carried out at the beginning of the rainy season (May 2012). Again, TRC volunteers conducted door-to-door visits to reinforce key standardized messages regarding the importance of using an ITN and its care but did so in five of the ten cantons that received the hang-up visit and the door-to-door visits in December (HU + 2 DTD). The final evaluation took place one month later, well into the beginning of the rainy season (June 2012).

A costing analysis was performed in 2011 adopting a providers’ perspective and using an ingredients approach to measure the incremental costs associated with a hang-up intervention following a LLIN distribution campaign. The analysis included only direct financial costs of managing and providing the intervention through the network of the TRC volunteers. Major drivers of cost identified were the 1) the costs of materials to hung the nets and transport it to the site of intervention (29%); and 2) the costs associated to the deployment of volunteers in the intervention area (47%). The overall cost of the intervention was $1.98 USD per household.

### Study objectives

Each evaluation survey was conducted to assess the effectiveness of the intervention most recently conducted and the sustained effectiveness of previous interventions. More specifically, the objective of the first survey was to evaluate the effectiveness of the hang-up visits conducted by TRC volunteers after the LLIN campaign in increasing ITN hang-up and utilization. Although the campaign provided LLINs to households, the standard Roll Back Malaria indicator refers to all ITNs as the basis for calculating population-level utilization of nets. Therefore, the analysis includes LLINs as well as other ITNs. The objective of the second survey was to evaluate the effectiveness of the dry season door-to-door visits conducted by TRC volunteers in increasing or sustaining ITN ownership and use as well as to assess any temporal effect the first intervention may have had on ITN ownership and use. Similarly, the objective of the final survey was to evaluate the effectiveness of the rainy season door-to-door visits conducted by TRC volunteers in increasing or sustaining ITN ownership and use as well as to assess any temporal effect the first and second interventions may have had on ITN ownership and use. In all three surveys, a comparison of ITN hang-up and utilization levels compared the intervention arms to the control arm of the study.

### Survey questionnaire

The survey questionnaire was developed based on the standard Malaria Indicator Survey (MIS) questionnaire in French and a questionnaire developed by NetWorks for a similar study in Uganda (Killian, pers comm). Information was collected on all nets owned by a household, all members and visitors of the household, and use of the nets and by whom. Additional information was collected on the demographic characteristics of the household including its geographic location; knowledge, attitudes and beliefs regarding malaria and net use; experience at the campaign distribution point and the intervention visits; and, nets recently eliminated from the household.

The questionnaire was programmed on personal digital assistants (PDAs; ASUS MyPal A696) using Visual CE (Syware, 2010). The questionnaire appeared on the PDAs in French but enumerators were trained with standardized translations of the questions from French into three local languages. The questionnaire was pre-tested in each local language in the study area in a non-participating community and minor changes were made to the questionnaire. Most questions were asked of the main respondent (normally the head of household or his/her spouse). Questions related to the campaign distribution point were asked only in the first survey and only of the person who had collected the LLINs.

### Sample size

Sample size calculations were made based on the pair-wise comparisons between intervention and control arms. The ICC (intraclass correlation) was estimated to be low (0.02) based on data from recent studies in Madagascar [15] and Malawi [16], as well as the relative socio-economic and cultural homogeneity of the study area.

Standard sample size calculations were first done for a two-sample comparison of proportions, α = 0.05 (2-sided), power of 90%. Based on previous estimates from Togo, a baseline ITN utilization prevalence of 50% was assumed, which is statistically conservative, as statistical power is lowest when the prevalence is closest to 50%. Power to detect a difference of 15% between intervention and control arms was targeted.

Based on these assumptions and testing various scenarios, a sampling design of 20 clusters (ten clusters per arm) with 46 households per cluster (total of 456 households per study arm) would power the study to observe a difference of 15%. Assuming a 5% non-response rate [17], a sample size of ten clusters per study arm was targeted with a simple random sample of 48 households per cluster (chosen from a complete list of household eligible for the LLIN campaign compiled from all villages), for a total sample of 480 households per arm and a total study sample of 1,440 households per survey. Sample size estimates were calculated using Stata 11 (Stata Corporation, Duxbury, USA).

### Sample size adjustments

Five communities that were assigned to the control cantons were visited in error by TRC volunteers during the first intervention period (post-campaign hang-up visits), due to recent changes in canton boundaries and supervisor error. Rather than exclude these communities from analysis, the number of households sampled in the three affected cantons was increased in the second and third evaluations. For each household randomly selected from a community that accidently received the intervention, an additional household was randomly selected outside that community within the same canton. This allowed the sample size of 48 households per control canton to be maintained while also surveying these five communities.

For the third evaluation, the third intervention arm (HU + DTD) was divided in half so that five of the ten cantons received an additional door-to-door visit in May at the beginning of the rainy season (HU + 2 DTD). To minimize loss of statistical power the number of households surveyed in arms HU + 1 DTD and HU + 2 DTD was increased. In total, 57 additional households were selected for each canton in arms HU + 1 DTD and HU + 2 DTD.

### Survey data collection

The names of the heads of the randomly selected households were obtained from the campaign registers. Each selected household was visited and the head of household or his/her spouse was interviewed. A replacement household was selected using standard methods (nearest neighbour) if the family had moved out of the area or would not be returning during the period of time that the survey team was in the area. In the first evaluation, three communities were deemed practically inaccessible and random replacements were chosen for these households. These three communities were eliminated from the sampling frame for the second and third evaluations. The third survey occurred during the rainy season when an additional 31 communities were deemed inaccessible and removed from the sampling frame. It is therefore preferable to interpret the results as representative of accessible communities in the study area, particularly for the final evaluation in June 2012.

### Statistical analyses

Data were downloaded from the PDAs to a Microsoft Access database where preliminary cleaning was done. The majority of cleaning involved corrections to the data as outlined by the enumerator in the Comments variable for each household. Where comments were not clear, the data were left unchanged. In the second evaluation, the child age in months calculation was incorrectly programmed in the PDA survey and was corrected post-hoc in data cleaning. Data analysis was conducted using the ‘survey’ package [18,19] in R, version 2.15.2 (R Core Team, 2012), Stata 11 and SAS (v9.3; SAS Institute, Cary, NC, USA). Analyses accounted for the clustered structure using PROC Surveyfreq and PROC SurveyLogistic in SAS or svyby and svyglm in R, weighted by inverse of the probability of household being selected and canton as the cluster. P-values are for Wald Chi-square. No adjustments were made for multiple comparisons in order to maximize statistical power.

Analysis used an ‘intention to treat’ approach, i e, all sampled households were included. The exception to this was that the five communities in the control cantons that inadvertently received the first intervention (the post-campaign hang-up visit) were re-assigned to the HU arm for all the analyses. The primary unit of observation was the household stratified by study arm. The primary comparisons were across the study arms – the control arm and the intervention arms (HU, HU + 1 DTD and HU + 2 DTD).

The effectiveness of the interventions was assessed based on their impact on four standard RBM indicators. The first indicator measured access to an ITN, defined as percentage of individuals of all ages with access to an ITN in their household, based on the assumption that each ITN covers two people. Three indicators were used to assess ITN use, including percentage of existing ITNs that were used the previous night, percentage of all individuals who slept under an ITN the previous night, and percentage of all children aged under five who slept under an ITN last night.

To explore the potential effect of household socioeconomic status on ITN access and utilization, each household’s socioeconomic status was assessed through a multiple correspondence analysis of household assets using Stata’s *mca* command. All individuals usually present in each household were assigned the household’s standardized wealth index score, and all individuals in the sample population were ranked according to that score. The sample population was then divided into quintiles of individuals, with all individuals in a single household being assigned to the same quintile. Three key indicators (ITN access, proportion of all individuals who used an ITN, and proportion of children under five who used an ITN) were related to wealth quintile for each evaluation to assess if varying wealth among intervention impacted the results and caused an imbalance among study arms. Access and use indicators were assessed by socioeconomic quintile using PROC surveyfreq and PROC surveylogistic in SAS to determine if any indicators varied significantly as a function of wealth. P values are for test of differences (using Rao-Scott chi-square goodness-of-fit tests) in indicator among all five wealth quintiles (overall) and tests of contrast between poorest quintiles (Q4 and 5) *versus* richest quintiles artiles (Q1 and 2) when the overall test was significant. A complete description of the multiple correspondence analysis can be found in Additional file 1.

### Ethics

Prior to the start of the study, the Togo Red Cross organized a one-day information and sensitization session in each district to inform political, administrative, health and traditional authorities about the operations’ research study objectives and activities planned. The protocol was reviewed and approved by the Togo Ministry of Health Bioethics Committee for Health Research and the HealthBridge Research Ethics Board. Prior to beginning the interviewers, free and informed consent was sought from the participants.

## Results

Data were available for a total of 1,427 of the 1,440 households selected for the sample in the first survey, 1,466 of 1,467 in the second survey and 1,555 of 1,557 in the third survey, for an overall response rate of 99.6% (there were three, zero and one refusals in the first, second and third surveys, respectively). Each study arm had a similar number of households unavailable that were replaced (14.8% replacements in first evaluation, 8.3% in second and 10.9% in third).

### Household characteristics by study arm

Household characteristics were similar across study arms and for all three surveys (Table 1). Approximately one-fifth of household heads were female. More than three-quarters of household heads had at least primary level education and 40% had also attended secondary school. Mean household size was approximately 4.5. Nearly half of households reported having at least one child under five years of age in the household.

**Table 1:** Household (HH) characteristics by study arm and evaluation

	**Study arm**					
**Characteristic**		**Overall**	**Control**	**HU**	**HU + 1 DTD**	**HU + 2 DTD**
N (# HHs)	Nov	1427	452	501	474	
	Jan	1466	474	513	479	
	Jun	1555	480	507	284	284
Female head of HH (%)	Nov	23.4	20.4	27.5	18.6	
	Jan	19.1	14.4	22.1	19.4	
	Jun	23	17.9	25.9	24	23.8
Heads with primary education (%)	Nov	73.5	79.1	65.1	84	
	Jan	79.6	83.4	76.5	80.2	
	Jun	79.4	81	76.9	81.3	80.9
Secondary education (%)	Nov	43	44.9	35.4	55.6	
	Jan	43	45.7	36.7	48.2	
	Jun	43.2	41.7	41.4	48.9	44.3
Mean HH size	Nov	4.6	4.6	4.6	4.8	
	Jan	4.2	4.3	4	4.3	
	Jun	4.3	4.5	4.4	4	4.2
HHs with > =1 child under 5 years (%)	Nov	48.5	56	43	51.4	
	Jan	47.3	53.6	45.3	44.6	
	Jun	46.3	51	44.2	42.1	47.7
Type of toilet						
Bush, open latrine, other (%)	Nov	73.6	80.6	72.7	68.4	
	Jan	76.8	79.8	77.1	74	
	Jun	76.3	84.4	73.9	59.2	86.3
Drinking water source						
Surface water (%)	Nov	53.5	77.7	39.1	56.6	
	Jan	65.2	77.4	56	66	
	Jun	61.3	77.8	52.3	55.7	61.1
Electricity (%)	Nov	26.8	10.5	34.4	28.5	
	Jan	17.3	9.7	20.8	19.2	
	Jun	17.4	8.6	19.2	29.7	15.2
Own land for farming (%)	Nov	51	58.5	47.7	49.9	
	Jan	55.7	57.5	55.2	54.8	
	Jun	58.8	63.7	56.1	59.2	56.5
Socioeconomic status						
Individuals in lowest wealth quintile (%)	Nov		20.5	20.4	19	
	Jan		14.9	18.1	26	
	Jun		24.2	18.7	14.4	20.4

Types of assets owned, access to electricity, type of cooking fuel, toilet and water source, as well as ownership of animals and land for farming were similar between arms and surveys. Three-quarters of households did not have sanitary toilet facilities and more than half used unprotected surface water for drinking. Less than one in four households had access to electricity and over half owned land for farming.

### Intervention coverage

Based on monitoring data collected during intervention implementation and analysed by TRC volunteers and staff, over 99% of households in the targeted communities were visited by volunteers (see Additional file 2). These records also indicate that 58% of nets were already hung during the first hang-up visit and the volunteers hung an additional 34%. By the second and third visits, the proportion of nets already hanging was 92 and 96%, respectively, and the additional nets hung during the visit was 1.3 and 0.8%.

From the household survey data collected during the evaluations, household recall of a volunteer visit in the intervention arms ranged from 80% for the hang-up visit to 75% for the first DTD visit and 64% for the second DTD visit. Over two-thirds of these households identified the person who visited as a Red Cross (i.e. Togo Red Cross) volunteer and the remainder as a community health worker. Consistent with the monitoring data, only one-quarter to one-third reported that volunteers assisted with the hanging of a net during the hang-up visits; approximately one-third of the households reported that the nets were already hung. During the first and second DTD visits, less than 10% of households were assisted with hanging a net. However, the majority of households receiving the first and second DTD visits reported that the volunteer provided new information. The most common answers for what new information was provided were: "how to care for the net", that "using a net is important for the prevention of malaria" and that it "should be used every night" and "by everyone in the household". In contrast, 23% of households who were not targeted to receive the hang-up intervention (those in the control arm) reported having received a visit from a volunteer, 77% of which reported that they were visited by the community health worker as opposed to a Red Cross volunteer. In the two subsequent evaluations, approximately 7% of households not targeted to receive the first or second DTD visit (those in the control and HU arms) reported having received a visit from either a Red Cross volunteer or a community health worker.

### ITN access indicators

**Figure 2:**
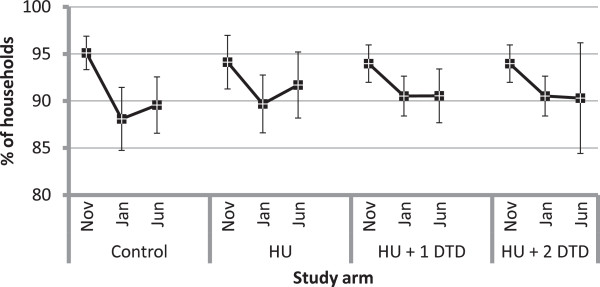
**Per cent of households that retained all campaign insecticide-treated nets received by study arm for each evaluation.** Within each study arm, the markers represent the estimates from the first (November 2011), second (January 2012) and third (June 2012) evaluations, respectively. Note that the results for the first and second evaluations are shared between the HU + 1 DTD and HU + 2 DTD arms and differ only for the third evaluation.

The national LLIN distribution campaign achieved high coverage in the study area. In the November evaluation, the levels of retention of campaign nets were high for all study arms, with over 95% of households reporting having kept all LLINs given. This decreased significantly in all arms between the November and January surveys (Control: Δ%_3–1_ = -7.0, p < 0.0001; HU: Δ%_3–1_ = -4.4, p = 0.005; HU + DTD: Δ%_3–1_ = -3.4, p < 0.0001; Figure 2) and then remained at similar levels between January and June. When asked why a campaign net was not retained, the majority (85 to 95% of respondents across all three study arms and all three surveys) said that someone else needed the net.

In the November evaluation, over 80% of households across arms met the criteria of having at least one ITN for every two people in the household, the goal set by the 2011 national campaign. The per cent of households with at least one ITN for every two people did not vary among arms in any survey but, as with campaign ITN retention, decreased significantly within each arm between November and January (Control: Δ_2–1_% = -10.9, p = 0.0002; HU: Δ%_2–1_ = -7.1, p = 0.047; HU + DTD: Δ%_2–1_ = -5.7, p =0.0012).

**Figure 3:**
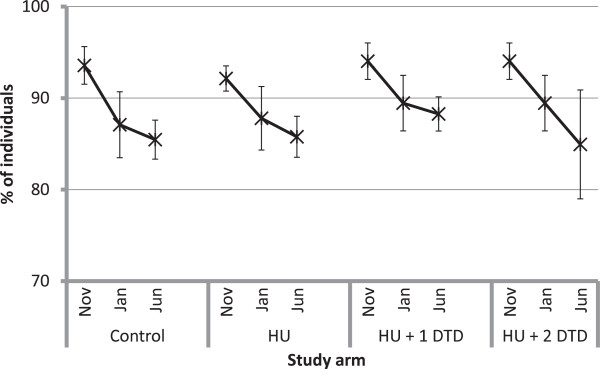
**The per cent of individuals with access to an insecticide-treated net (if one ITN covers two people) by study arm.** Within each study arm, the markers represent the estimates from the first (November 2011), second (January 2012) and third (June 2012) evaluations, respectively. Note that the results for the first and second evaluations are shared between the HU + 1 DTD and HU + 2 DTD arms and differ only for the third evaluation.

The per cent of individuals with access to an ITN (assuming that one ITN covers two people) followed a similar trend to that detected in the percent of households that retained all campaign nets (Figure 3). It did not differ significantly among study arms within surveys but generally tended to decrease significantly between the November and June surveys within study arms (Control: Δ%_3–1_ = -8.1, p < 0.0001; HU: Δ%_3–1_ = -6.4, p < 0.0001; HU + 1DTD: Δ%_3–1_ = -5.8, p = 0.001; HU + 2DTD: Δ%_3–1_ = -9.1, p < 0.0001).

### ITN utilization

**Figure 4:**
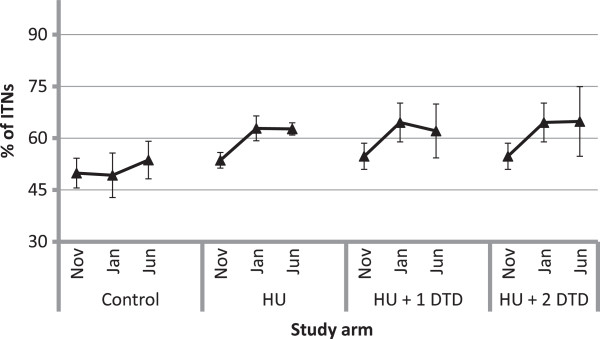
**Proportion of existing insecticide-treated nets in the households used the previous night.** Within each study arm, the markers represent the estimates from the first (November 2011), second (January 2012) and third (June 2012) evaluations, respectively. Note that the results for the first and second evaluations are shared between the HU + 1 DTD and HU + 2 DTD arms and differ only for the third evaluation.

Various measures of ITN utilization were assessed by study arm and by evaluation, with results shown in Table 2. Approximately 50% of existing ITNs were used the previous night across all study arms in November (Figure 4). Use of ITNs was about 10% higher in January in the intervention but not the control communities (HU: Δ%_2–1_ = 9.3, p = 0.009; HU + DTD: Δ%_2–1_ = 9.8, p = 0.009). No change in use was observed in any study arm between January and June.

**Table 2:** Weighted frequencies and 95% confidence intervals for insecticide-treated net utilization indicators

	**Study arm**											
	**Control**			**HU**			**HU + DTD**				**Overall**	
	**N**	**%**	**95% CI**	**N**	**%**	**95% CI**		**N**	**%**	**95% CI**	**%**	**95% CI**
**% of existing ITNs used the previous night**												
Nov	1336	49.9	45.6, 54.2	1384	53.6	51.3, 55.9		1345	54.8	51.0, 58.5	53.0	51.4, 54.6
Jan	1185	49.2	42.8, 55.7	1210	62.8*	59.2, 66.4		1166	64.5*	58.9, 70.2	59.6	55.2, 64.1
June	1148	53.7	48.2, 59.1	1225	62.7*	60.9, 64.5	1 DTD	646	62.1	54.3, 69.9	60.4	57.0, 63.8
							2 DTD	640	64.8*	54.8, 74.9		
**% of individuals who slept under an ITN the previous night**												
Nov	1963	70.2	64.4, 75.9	2205	67.0	60.6, 73.3		2064	69.7	65.5, 73.9	68.5	64.2, 72.8
Jan	1949	65.9	56.8, 74.9	2034	76.7*	73.7, 79.7		1864	79.5*	72.6, 86.3	74.6	70.4, 78.9
June	2050	66.0	56.0, 75.9	2177	70.5	66.6, 74.4	1 DTD	1011	71.4	65.8, 77.0	70.4	66.5, 74.4
							2 DTD	1162	**77.2**	71.1, 83.4		
**% of children <5 years who slept under an ITN the previous night**												
Nov	354	79.1	75.6, 82.6	350	76.9	70.4, 83.4		345	82.9	80.0, 85.9	79.2	75.3, 83.2
Jan	321	74.2	67.3, 81.1	363	80.6	75.0, 86.2		310	**87.0**	79.6, 94.5	80.7	76.3, 85.1
June	353	75.5	70.0, 81.0	353	82.6	73.7, 91.6	1 DTD	166	**82.2**	80.8, 83.6	81.6	77.0, 86.2
							2 DTD	195	**90.0**	84.4, 95.5		
**% of WRA (15–49 years) who slept under an ITN the previous night**												
Nov	429	74.1	69.4, 78.9	487	68.5	60.7, 76.2		459	71.9	68.4, 77.9	71.0	65.1, 76.9
Jan	430	68.9	59.3, 78.5	433	79.4*	74.0, 84.7		429	79.9	70.7, 89.1	76.6	71.6, 81.5
June	449	66.7	55.3, 78.2	468	72.6	68.2, 77.1	1 DTD	243	73.2	63.0, 83.5	72.4	67.6, 77.3
							2 DTD	269	81.0*	75.3, 86.6		

ITN refers to an insecticide-treated net.

WRA refers to women of reproductive age.

Four key indicators are presented overall and by study arm and evaluation with their associated sample size (N) and 95% confidence intervals (95% CI). Indicators in the intervention arms that are significantly different from the control arm are highlighted in bold. Indicators that are significantly different from the November survey within study arms are indicated with an asterisk.

**Figure 5:**
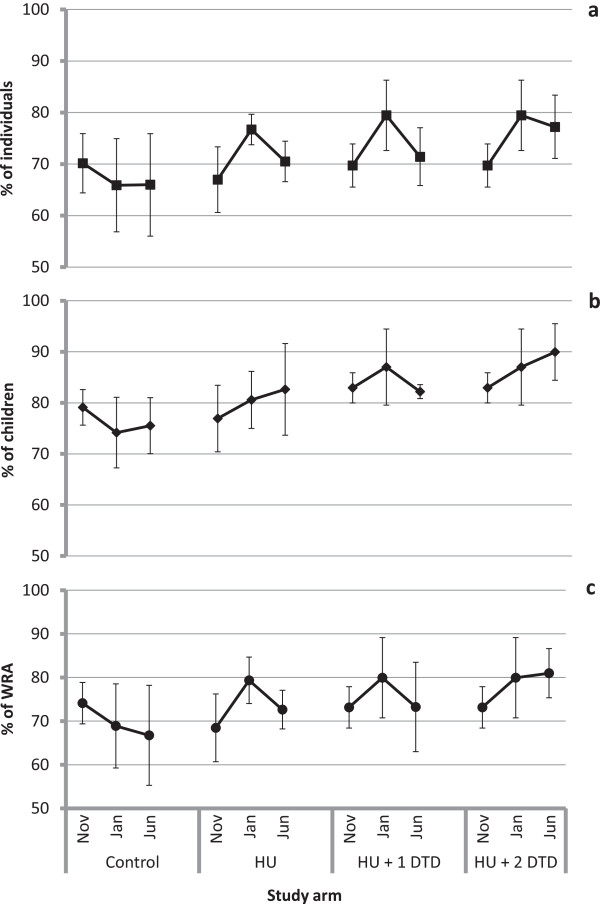
**The per cent of individuals reported to have slept under an insecticide-treated net the previous night for each of the study arms.** Panel **a** represents the per cent of all individuals, **b** is the per cent of children under five years and **c** is the per cent of women of reproductive age (18–49 years). Within each study arm, the markers represent the estimates from the first (November 2011), second (January 2012) and third (June 2012) evaluations, respectively. Note that the results for the first and second evaluations are shared between the HU + 1 DTD and HU + 2 DTD arms and differ only for the third evaluation.

In terms of utilization by individuals, in the November evaluation 68.5% of individuals slept under an ITN the previous night overall, with similar levels across all three study arms (Figure 5a). This rose to 74.6% overall in the January evaluation but trends within study arms were quite different. In the control arm, the per cent of individuals using an ITN remained similar between the November and January evaluations; however, in the intervention arms, individual use increased (HU: Δ%_2–1_ = 9.7, p < 0.0001; HU + DTD: Δ%_2–1_ = 9.8, p < 0.0001) and was greater than in the control arm (HU: Δ%_HU-Control_ = 10.8, p = 0.025; HU + DTD: Δ%_HU+DTD-Control_ = 13.6, p = 0.006). By the June evaluation, individual use declined in all intervention arms, though less so in the HU + 2DTD, which had received the most recent intervention and where use remained significantly greater than in the November survey (HU: Δ%_3–2_ = -6.2, p = 0.001; HU + 1DTD: Δ%_3–2_ = -8.1, p = 0.006; HU + 2DTD: Δ%_3–2_ = -2.3, p = 0.020; HU + 2DTD: Δ%_3–1_ = 7.5, p = 0.073). In both the January and June evaluations, the study arm that had received the most recent intervention had significantly greater individual use of ITNs than the control arm (January: Δ%_HU+DTD-Control_ = 13.6, p = 0.006; June: Δ%_HU+2DTD-Control_ = 11.2, p = 0.042).Following the campaign, the proportion of children under five years who slept under an ITN the previous night was slightly higher than for all individuals, at 79.2% overall and also similar across study arms, ranging from 79.1 to 82.9% (Figure 5b). Use by children remained constant in the control arm over the three evaluations (November: 79.1 ± 3.5%; January: 74.2 ± 6.9%; June: 75.5 ± 5.5%). In the dry season, use of ITNs by children under five was 12.8 percentage points higher (p = 0.022) among households in the arm that received the first two interventions compared to households in the control arm. In the rainy season, use of ITNs by children was 6.7 percentage points higher (p = 0.008) in the arm that received only the first two interventions and 14.5 percentage points higher (p = 0.001) in the arm that received all three interventions.

In November, 71% of women of reproductive age (WRA; 15–49 years) slept under an ITN the previous night, with similar levels in each study arm (Figure 5c). Similar to children under five, ITN use by WRA increased between November and January by as much as 10 percentage points (HU: Δ%_2–1_ = 10.9, p = 0.0002) but returned to November levels by June in all but the study arm that received the last intervention. In June, use by WRA in the HU + 2 DTD arm was 14.3 percentage points greater than in the control communities (p = 0.015).

With one exception, ITN access and use was unrelated to wealth quintile. In the November evaluation, ITN use by all individuals differed significantly among wealth quintiles and was significantly higher in the two poorest quintiles than the two wealthiest quintiles (Q4&5: 74.4%, Q1&2: 67.6%, p = 0.003; see Additional file 1). ITN access, ITN use by all individuals and ITN use by children under five was unrelated to wealth quintile in all other evaluations. Therefore it is unlikely that a wealth imbalance among study arms contributed importantly to the observed results.

### Reported reasons for non-use

Reasons for not using a specific net the previous night were similar across study arms (see Table 3). In November, about one-third of the unused nets were reported to be in excess of the current needs of the household, i e, everyone in the household was already sleeping under a net and therefore the net was not utilized. This decreased to approximately 20% in the subsequent evaluation. Anecdotal evidence indicates the drop between November and January occurred because students studying outside the community returned home for Christmas and took campaign nets with them when returning to school, particularly those studying in Lomé where LLINs were not distributed during the campaign. Twenty-eight per cent of non-used nets were reportedly unhung due to a lack of space, material or knowledge necessary to hang the net. This decreased to 5.4 and 2.9%, respectively, in subsequent surveys. The most common reason for not using a specific net in the January and June evaluations shifted to "Not yet hung up/kept as a reserve net". Few nets (2.9%) were not used due to a negative experience with them.

**Table 3:** Reasons for non-use

**Reason given by respondent**		**Study arm**				**Total**
		**Control**	**HU**	**HU + 1 DTD**	**HU + 2 DTD**	
N	Nov	710	576	563		1,849
	Jan	607	425	403		1,435
	Jun	498	459	239	203	1,399
All already sleeping under a net	Nov	37.0	29.3	35.3		33.5
	Jan	18.3	20.9	20.1		19.6
	Jun	17.7	15.9	17.2	27.1	18.4
Unable to hang (including no place, materials or knowledge)^1^	Nov	23.8	32.5	26.8		28.1
	Jan	6.9	4.0	4.5		5.4
	Jun	3.4	2.6	2.5	3.0	2.9
Too damaged or dirty	Nov	7.4	11.2	11.4		10.1
	Jan	5.1	6.6	10.9		7.2
	Jun	5.2	5.4	5.4	8.4	5.8
Normal user absent/reserved for guests^2^	Nov	10.2	8.9	8.2		9.1
	Jan	6.6	10.6	10.7		8.9
	Jun	10.0	10.7	15.1	11.8	11.4
Too hot/no mosquitoes/no malaria	Nov	8.3	6.7	10.1		8.3
NB: asked only of people in the 2nd survey	Jan	N/A	N/A	N/A		N/A
	Jun	4.2	5.0	7.5	5.9	5.3
Negative experience	Nov	2.1	4.6	1.6		2.9
NB: asked only of people after 1st survey						
Not yet hung up/kept as a reserve net^3^	Nov	1.9	2.4	1.6		2.0
	Jan	30.5	25.2	26.3		27.7
	Jun	25.3	26.6	25.1	19.7	24.9
Replaced by a new one^3^	Nov	0.8	0.2	1.1		0.7
	Jan	6.4	8.9	10.2		8.2
	Jun	12.2	15.0	18.4	11.8	14.2
Currently being washed or dried^3^	Nov	0.6	0.4	0.3		0.4
	Jan	5.6	5.9	3.0		4.9
	Jun	7.2	9.2	2.5	6.9	7.0
User has moved or given to someone else^4^	Nov	1.8	1.1	0.6		1.1
Temporarily outside of the household^4^	Nov	0.6	0.0	0,2		0.3
Used for other purposes^5^	Nov	2.7	1.7	1.4		1.9
Don’t know	Nov	2.5	0.3	0.0		0.9
NB: no one responded "Don’t know" in 2nd survey	Jan	0.0	0.0	0.0		0.0
	Jun	2.2	0.4	0.8	1.5	1.3
Other^6^	Nov	0.2	0.8	1.5		0.8
	Jan	20.6	17.9	14.4		18.0
	Jun	12.4	9.2	5.4	3.9	8.9

^1^Original category "Nowhere to hang" was expanded to include all aspects of "Unable to hang" reported when the enumerator specified "Other" in the first survey.

^2^Original category "Normal user absent" was combined with "kept for company", which was often reported when the enumerator specified "Other" in the first survey.

^3^New category created and kept for subsequent surveys based on the answers frequently given when specifying "Other" in the first survey.

^4^New category created based on the answers frequently given when specifying "Other" in the first survey but not included as a separate category for subsequent surveys.

^5^Not included as a separate category for subsequent surveys.

^6^ For the first survey, "Other" excludes the answers combined into new categories.

The relative proportions of answers given for the main reason why a particular net was not used or why a particular person did not use a net the previous night are provided below. Proportions (unweighted) are given by study arm for each evaluation.

**Figure 6:**
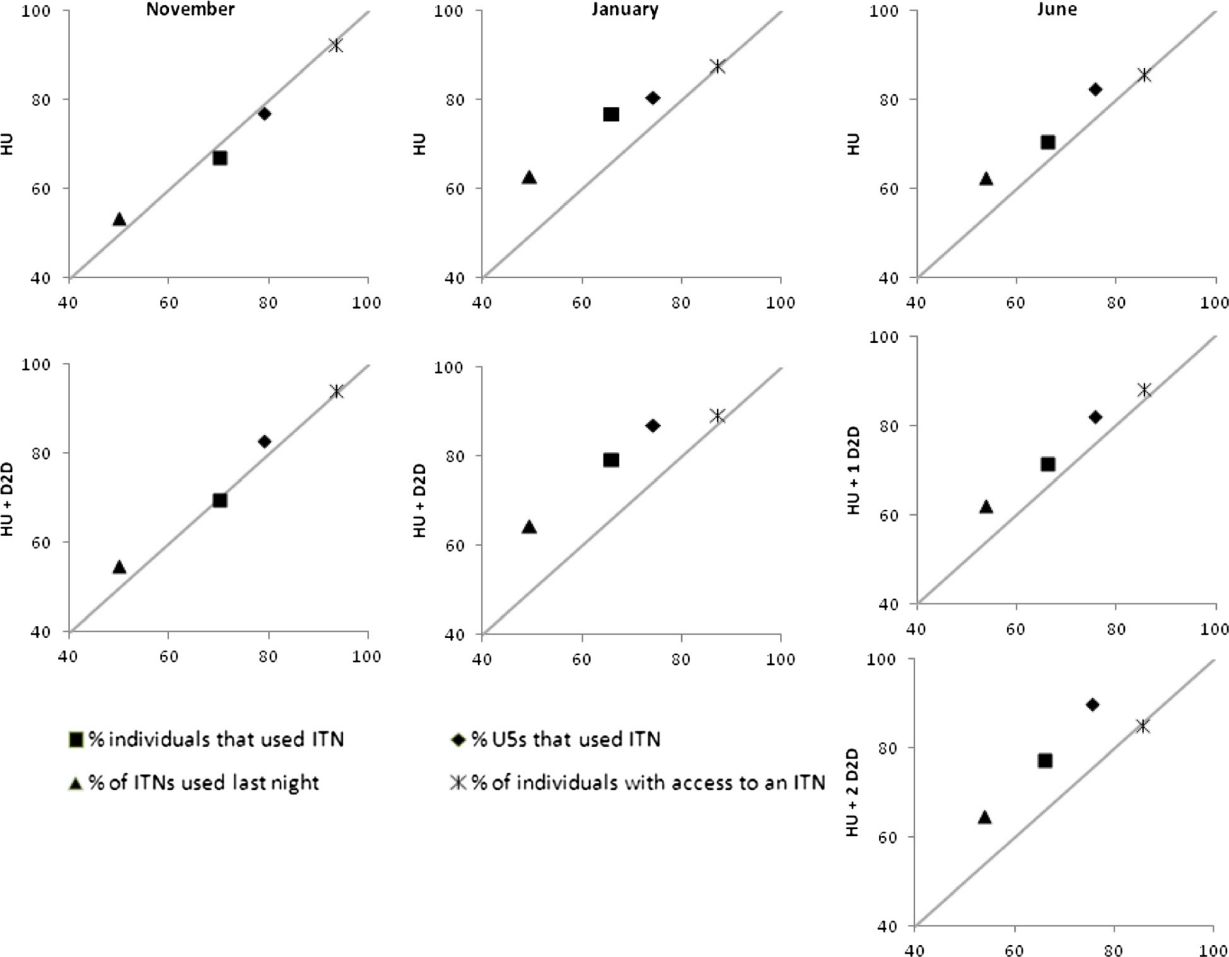
**Scatterplot of access (star) and use (solid) indicators in HU and HU + DTD intervention arms on y-axis *versus *control arm (on the X-axis).** Communities in the HU arm received only the hang-up visit immediately following the mass distribution campaign in October 2011. The communities of the HU + DTD arm received a follow-up door-to-door visit in December 2011 and the communities in the HU + 2 DTD arm (one half of HU + DTD) received a second follow-up door-to = door visit in May 2012.

In the January and June evaluations, respondents were asked why a given person did not use a net in addition to asking why a net was not used see (Additional file 3). The most common reason overall for not having slept under a net the previous night was an insufficient number of nets ("Not enough nets"). Less common answers, especially in the rainy season, were that it was too hot, there were no mosquitoes or they had had a negative experience regarding use of a net. Among households with fewer than one ITN per two people, more than half of those that did not sleep under a net the previous night failed to do so because there were not enough nets. However, even in households with sufficient ITNs (71.7-83.8% of all households), 13 to 20% of individuals (depending on the evaluation) who did not sleep under a net the previous night were reported to have not done so due to insufficient nets. In general, individuals in households with sufficient nets were more likely to cite that it was too hot, there were no mosquitoes, or a negative experience as reasons for not having used a net the previous night than individuals in households with an insufficient number of nets. A large proportion (39-44%) of responses fell into the category "Other", suggesting that there are important reasons for lack of use that were not considered in this study.Figure 6 depicts the change in the access indicator (shown with a star symbol) and use indicators (shown with solid symbols) for each intervention arm relative to the control arm over the three evaluations. Estimates falling on the 1:1 line cutting diagonally upward indicate that there is no difference between control and intervention. Estimates that fall above the 1:1 line indicate that the intervention resulted in higher indicators of access or use. Moving from left to right, the progress from early in the study (November) to the end of the study (June) is evident, with access remaining near the line and use increasing over time. Similarly, moving from top to bottom within surveys, the larger impact of the most recent intervention is marked looking at the graphs for least amount of intervention (HU arm with only one visit) to the most intervention (HU + 2 DTD with three visits).

## Discussion

ITN use was fairly high in this region of Togo where as many as 70% of all individuals and 79% of children under five years were reported to have used an ITN the previous night. Measured in the control arm communities in each evaluation, ITN utilization did not vary significantly over the course of the study, decreasing slightly from 70 to 66% for all individuals taken together and 79 to 74% by children under five years. While still below the WHO target of 80% use by all individuals, and children under five and pregnant women in particular [3], these levels are greater than those observed after the LLIN distribution campaign in 2004 [20] and consistent with what was observed after the LLIN distribution campaign in 2008 [9]. These reasonably high levels of ITN use suggest that the Plateaux region of Togo is instilled with a sound net culture.

In general, households that received intervention visits, particularly the most recent intervention visit, had levels of use that were 5 to 10% higher than the control households while access did not differ among control and intervention households. In the final evaluation in June, ITN use by all individuals, children under five and women of reproductive age was 11.3 to 14.4 percentage points greater in the HU + 2DTD arm, which received all three interventions, than the control communities. It was hypothesized that the interventions would lead to a minimum increase in ITN use of 15%. However, increases of the magnitude observed here as a result of hang-up interventions are consistent with what has been previously observed in Togo when use by the general population (as estimated in the control communities) is moderate (approximately 70%). In Togo, after their first mass distribution campaign of LLINs, it was found that ITN utilization was significantly higher in households that received a follow-up visit compared to those that did not, increasing from 72 to 80% [20]. The survey conducted in Togo following the second mass distribution campaign found that baseline ITN utilization was low (54%) and showed a much larger increase in this case (24 percentage point difference) in the proportion of nets hanging eight months after a campaign when comparing households that received (78%) a ‘hang-up’ visit [9]. Elsewhere, increases in net utilization after hang-up interventions are often much lower. In Niger, ITN use by children under five only increased by 3 percentage points after intervention by Red Cross volunteers [21]; in Madagascar an increase of 5 percentage points (from 90-95%) was reported [22]. One major difference between this study and the Niger study was the coverage achieved by the intervention. In Niger, only 20.2% of households reported that a Red Cross volunteer visited their village and only 6.5% reported that one visited their household. In this study, visits were reported by 64 to 80% of households in intervention areas.

The magnitude of the differences in levels of ITN use between the control and intervention households increased considerably between the November and January evaluations. In November, households that had received hang-up visits showed only small differences in levels of ITN use compared to households that did not receive these visits, with slightly higher levels of use by children under five. However, in the January evaluation, the magnitude of the differences between the intervention and control communities for ITN use indicators increased to as much as 13.7 percentage points (Table 2). This occurred regardless of whether or not the households had received only the post-campaign hang-up visit (HU) or the households had also received the dry season door-to-door visit (HU + DTD), although use tended to be higher in the households that had received the second intervention as well (Table 2). An increase in ITN use over time since a mass distribution campaign, regardless of whether or not households had received hang-up interventions, was also observed in a parallel study conducted recently in Uganda (Killian, pers comm). This could result partly from a lagged response to increased awareness of malaria and ITN use generated by the mass distribution campaign. Thwing *et al.*[21] also observed increases in ITN use as time passed after a nationwide integrated campaign in Niger. However, in both the Niger and Uganda studies, the increases in ITN use were confounded by transitions to increasingly wet weather and therefore increasing presence of mosquitoes. This was not the case in this study where the increase in ITN use occurred predominantly during the dry season, with little increase observed during the rainy season.

The reason for the increase in ITN use in the intervention arms appears to be due to the novelty of the distributed ITNs combined with a mass effect. A qualitative study was conducted in parallel to the quantitative study, aiming to determine the factors of non-use and use of ITNs in the Plateaux Region [23]. Results from the qualitative study suggest that the reason for the increased use of ITNs over the three months after the campaign was the motivating effect of the novelty of the ITNs combined with a mass effect, where sufficient individuals adopted the behaviour (using an ITN) to motivate others to also adopt the behaviour, as a result of the majority of households having and using new ITNs. Hanging an ITN became fashionable as a consequence, particularly because, with their blue colour, they were considered a desirable element to add to a room. The increase in visits by friends and family over the holiday season in December, and consequently increased discussion in general, may have also contributed to this effect.

ITN use decreased between the January and June surveys in all study arms except in the households that received the most recent intervention (HU + 2 DTD), the May door-to-door visit, where use tended to remain stable or increase. The decline in use occurred despite the transition from the dry season to a wetter than average rainy season [24], when nuisance biting is expected to be higher [25]. This is in contrast to the findings of Killian *et al.* (pers comm) that found that ITN use continued to increase to the end of the study. Notwithstanding this decrease (in all but the HU + 2 DTD arm), ITN use did not decline further than the levels observed in November. This is reassuring given that this level of use is still greater than that measured in the 2010 MIS prior to this most recent LLIN distribution campaign. The decrease in ITN use in all communities except those that received the May door-to-door visit, combined with the finding above that even households that had already received two visits still reported acquiring new information from the volunteer, suggests that regular delivery of messages are important to sustaining use after a campaign.

ITN use by children under five years was higher than use by all individuals taken together in all study arms and all surveys. Two previous evaluations of ITN use in Togo following mass distributions of LLINs (targeting children under five years in both cases) reported only ITN use by children and pregnant women [9,20]. It is therefore not possible to say if higher use by children is reflective of customary within-household patterns of use in Togo or if it is due in part to the legacy of the two previous mass distribution campaigns that emphasized the use of ITNs by children under five years. However, in their evaluations of ITN use in Madagascar following its mass distribution campaign in 2007, Kulkarni *et al.*[26] found that use of ITNs by children under five years was substantially greater than that by all individuals taken together (80.8% of children under five years used an LLIN the previous night compared to 59.9% when all individuals were considered). This suggests that higher levels of use by children in this study may be reflective of within-household patterns of ITN use that are typical of other areas as well.

### Discussion of determinants of ITN use

Due to the high level of coverage achieved by the campaign, ITN ownership likely does not explain variability in ITN use in this study given that it does not vary across arms over time. There was little evidence of inequity in the study samples based on analysis of outcomes by household socioeconomic status (see Additional file 1). This is consistent with other studies that have shown that socioeconomic status may contribute to household ITN ownership but once a household owns a ITN, socio-economic status is not associated with ITN use [7,27].

A recent review of barriers to mosquito net use in malaria-endemic countries found that personal discomfort and perceived low mosquito density were the most common reasons for non-use [28]. In this study, these reasons were not frequently cited. The most common response for why nets were not used was that all individuals were already using nets, suggesting that surplus nets were now available in these households. Conversely, the most commonly given reason for why a person did not use a net was an insufficient number of nets in the household, particularly in households with fewer than one ITN per two people (56.3 *versus* 13.4% in households with at least one ITN per two people), suggesting that while access was high throughout the study area, access remained a barrier to ITN use for some. In households with at least one ITN per two people, "Not enough nets" was still the most common response in the January evaluation (13.4%). This is interesting because it indicates that even among households that are presumed to have enough ITNs for all its inhabitants, it was perceived that as much as 20% of the individuals did not have access to a net.

In the first evaluation post-campaign (November), the second most common response for not using a net was related to technical aspects of hanging the net, including no bed, materials or knowledge, and this did not vary across study arm. Small-scale, community-based interventions have been shown to effectively address this barrier in some contexts [29]. Anecdotal evidence from the field during the November evaluation as well as findings from the qualitative study [23] suggest that the common use of traditional mats on the floor (as opposed to beds) and limited space for sleeping continue to be barriers to ITN use, given the difficulty in hanging the net each night and the risk of damage to nets caused by the material of the mats. However, the proportion of households reporting this response for non-use of a net dropped considerably in the second and third evaluations with little difference observed between the control and intervention communities (November: 28.1%; January: 5.4%; June: 2.9%) suggesting that given time, individuals were able to find solutions to the difficulties with hanging.

It appears that the impact of the interventions was primarily through the delivery and reinforcement of key BCC messages regarding the importance of using an ITN and its care. ITN use was higher among households reporting that they observed a demonstration of hanging up an ITN at the campaign distribution point and among households where the person attending the distribution recalled the main message as "sleep under net every night". In households that received one (HU + 1 DTD) or two (HU + 2 DTD) additional door-to-door visits, the majority of respondents indicated that the volunteer provided new information during his/her visit regarding the use and importance of ITNs (72% and 58%, respectively) despite having received multiple visits, for which TRC volunteers were trained to deliver the same standardized key BCC messages each time. Regardless of whether the respondents in fact received new information or had forgotten earlier information, this suggests that regular visits from community agents are useful in reinforcing key BCC messages. Despite the fact that one central goal of the intervention was to physically assist households with the hanging of ITNs to encourage ITN use, it seems that the principal utility of the house-to-house visits was the regular messaging of the importance of using an ITN. The apparent effectiveness of the BCC strategies implemented as part of the national campaign provides evidence that ITN utilization also may be increased, at least in the short-term, using interventions that are less resource-intensive than house-to-house visits.

### Study limitations

The potential for dilution of the intervention effect cannot be ruled out in this study. Intervention and control clusters (cantons) bordered each other and, in some cases, shared the same primary health care unit. This may have contributed to informal sharing of the intervention messages. In addition, a small proportion of control households received the first hang-up intervention in error. It is unclear why approximately 20% of control arm households reported having received a visit from a volunteer, even though this could not be verified to be a visit from a study volunteer. A small proportion of visits in control areas were reportedly done by TRC volunteers but this may have been due to the fact that many community health volunteers are also TRC volunteers and may have been wearing Red Cross-identifying articles of clothing. The study arguably did not have a "true" control where control household would have received visits that were unrelated to malaria or ITN use. Consequently, it is possible that the visit itself rather than the messages delivered during the visit led to the observed effect.

Statistical power played an important role in this analysis. The study was powered to detect a difference of 15% in ITN utilization between intervention and control arms; however, based on the effect size observed in this context (5-10% rather than 15%), the study was under-powered. Moreover, the ICC for the main outcome indicators was greater than initially predicted (initial prediction: 0.02) for most of the evaluations. This will have further reduced the ability to detect statistically significant differences [14].

Prior to the November evaluation, households in the study area may have been alerted by volunteers that an evaluation team was coming to assess ITN hang-up and use, as reported anecdotally by the survey enumerators. It is not known to what extent this may have affected the results. If this was a factor, it would have been expected to increase ITN hang-up and utilization (or reported utilization) in the intervention cantons and inflate the effect of the hang-up visits. Given that use increased in these areas in the subsequent evaluation, it is unlikely that there was much of an effect.

Although every effort was made to ensure random sampling of households, a small percentage (~5%) of errors were found in the reported number of households in the community registers on which the sampling frame was based. The sampling frame was updated prior to each new survey, as more accurate information became available. Problems with accessibility of a small number of communities, particularly during the rainy season (June survey), may have biased the sample away from small, isolated communities. Finally, due to the timing of the first survey during the harvest season, enumerators reported difficulties in accessing household members during the day. Although reasonable efforts were made to administer the survey to the randomly selected household, replacement rates were higher than expected, although similar across study arms.

Contextual factors, including the fact that this was the third mass distribution of LLINs in Togo and there was a pre-existing net use culture in the study districts, may have played an important role in ITN utilization by households and individuals. The effectiveness of high-intensity interventions, such as hang-up and door-to-door visits, may not be as high in this type of setting as would be observed elsewhere.

## Conclusions

One month following the distribution of LLIN and implementation of the hang-up visits, little effect of the hang-up visits on ITN use was observed. ITN access and use were high across study arms including the control arm. However, ITN utilization typically declined in the intervening months between the dry season evaluation (January) and the rainy season evaluation (June) except in the communities that received the most recent intervention, the door-to-door visit in May, where ITN use remained stable or increased slightly. This, combined with the reporting by many in these communities that the TRC volunteers provided new information in the most recent visit, suggests that regular visits from community agents are useful in reinforcing key BCC messages and maintaining ITN use. It may be beneficial to explore more easily sustained methods of regularly reinforcing key messages such as SMS (short message service or "text") messaging, which has been used effectively for delivering health BCC interventions [30], including in sub-Saharan Africa [31].

## Competing interests

The door-to-door interventions were implemented in the field by the Togolese Red Cross and were funded by the International Federation of Red Cross and Red Crescent Societies (IFRC). HealthBridge and URD were funded by IFRC to conduct the quantitative evaluation of the impact of the intervention.

## Authors’ contributions

KS, PRB, KB, and GKB developed the original conception of the study; KB and GKB implemented the intervention in the field; RED, KS, DG, and KV led the data collection; RED, KS and PRB analysed the data; AS led the conception of and conducted the costing analysis; RED and KS wrote the first draft of the manuscript; and all authors contributed to revisions. All authors read and approved the final manuscript.

## Supplementary Material

Additional file 1 **"Wealth" index development using multiple correspondence analysis and impact of socioeconomic status on ITN access and use.** Description of the data analysis of the household characteristics to obtain a wealth score using multiple correspondence analysis (MCA) and results for the analysis of access and ITN use by household wealth, as determined by quintile membership. A socio-economic score was allocated to each household using a multiple correspondence analysis of household assets. Households were divided into five equal groups (quintiles) according to their level of wealth, and by definition approximately 20% were in each quintile. Quintile 1 represented the richest households and quintile 5 the poorest households. Each access and use indicator was assessed by socioeconomic quintile using PROC surveyfreq and PROC surveylogistic in SAS to determine if any indicators varied significantly as a function of wealth. P values are for test of differences (using Rao-Scott chi-square goodness-of-fit tests) in indicator among all five wealth quintiles (overall) and tests of contrast between poorest quintiles (Q4 and 5) *versus* richest quintiles (Q1 and 2) when the overall test was significant. Click here for file

Additional file 2 **Hang-up and door-to-door visit monitoring results from the study area cantons as reported by volunteers.** Description of data: Monitoring data collected by Togo Red Cross volunteers and staff during the implementation of the three phases of the hang-up interventions. Data provided by Karen Bramhill, the IFRC Operations Research Delegate overseeing the intervention implementation, and Ben Adinoyi, the Africa Health and Care Coordinator at IFRC. Click here for file

Additional file 3 **Reasons for non-use by household access.** Description of data: Reasons cited why a person did not use a net the previous night by study arm (unweighted) for households with sufficient access to ITNs (at least one ITN per two people) and without for the second and third evaluations (January and June 2012). Click here for file
