# Supplementary material for: Effectiveness of post-campaign, door-to-door, hang-up, and communication interventions to increase long-lasting, insecticidal bed net utilization in Togo (2011–2012): a cluster randomized, control trial
Source: Malar J. 2014 Jul 9;13:260. doi: 10.1186/1475-2875-13-260 (PMC4110632; doi:10.1186/1475-2875-13-260)
Supplement: Additional file 1 — "Wealth" index development using multiple correspondence analysis and impact of socioeconomic status on ITN access and use. Description of the data analysis of the household characteristics to obtain a wealth score using multiple correspondence analysis (MCA) and results for the analysis of access and ITN use by household wealth, as determined by quintile membership. A socio-economic score was allocated to each household using a multiple correspondence analysis of household assets. Households were divided into five equal groups (quintiles) according to their level of wealth, and by definition approximately 20% were in each quintile. Quintile 1 represented the richest households and quintile 5 the poorest households. Each access and use indicator was assessed by socioeconomic quintile using PROC surveyfreq and PROC surveylogistic in SAS to determine if any indicators varied significantly as a function of wealth. P values are for test of differences (using Rao-Scott chi-square goodness-of-fit tests) in indicator among all five wealth quintiles (overall) and tests of contrast between poorest quintiles (Q4 and 5) versus richest quintiles (Q1 and 2) when the overall test was significant. [file 1475-2875-13-260-S1.pdf]

Additional file 1 – "Wealth" index development using multiple correspondence analysis and impact of socioeconomic status on ITN access and use.

Background

It is our intention to explore the potential effect of household socioeconomic status on bednet utilization and malaria-related knowledge and practices. Given the difficulty in measuring household income or consumption expenditure due to recall bias, seasonality and data collection burden, asset-based measures are often used as a proxy for longer-term household economic status (Vyas and Kumaranayake, 2006). Following collection of asset variables at the household level, it is necessary to aggregate this data in some way since most individual asset variables are not sufficient to differentiate household socioeconomic status (Vyas and Kumaranayake, 2006). Filmer and Pritchett (2001) propose that the use of Principal Components Analysis (PCA) is pragmatic, avoiding the problems associated with using equal weights for all variables in the index or attempting to estimate the price or value of each asset. However, PCA was designed for use with continuous, normally-distributed variables and therefore its application to the categorical variables in a wealth index is considered by some to be inappropriate (Booysen et al., 2008; Howe et al., 2008). Multiple Correspondence Analysis (MCA) is analogous to PCA but is designed for use with discrete data. Based on work by Howe et al. (2008), comparison of indices generated by PCA and MCA showed a high level of agreement.

Methods

Vyas and Kumaranayake (2006) propose four key steps in constructing a socio-economic status (SES) index. The first is the selection of asset variables to be included in the index; the second is application of PCA (MCA in this case) to extract the first principal component (or dimension); the third is interpretation of results and creating the new variable using the weights from the first principal component; and finally, the fourth step is classifying household into socio-economic groups. These steps were applied to the Togo survey data set in order to construct a proxy variable for household wealth.

Step 1: Selection of asset variables

The Togo survey collected a variety of information related to the household's socioeconomic status, including ownership of durable assets, housing characteristics and access to sanitation facilities and source of water. Most of these variables are consistent with the wealth index developed by the World Bank for the Togo Demographic and Health Survey (DHS) 1998. See Table 1 below for a list of the potential asset variables and their distribution in the November 2011 dataset.

Determining the best combination of variables to include in the index:

1. Compare factor scores of different variable groups (e.g. durable assets, housing characteristics, water/sanitation access, agricultural variables)
2. Look at the distribution of household index scores using different variable groups to assess level of clumping and truncation

For several of the housing material and water/sanitation variables, similar responses were combined to create a smaller list of variables to be entered into the model.

Exploration of using different groups of variables in the model showed that the maximal variation explained by the first dimension and best distribution of scores across the continuum was achieved by including only the major assets owned (excluded fridge, landline, stove, fan, gas lamp, car & tractor, due to very low frequency of ownership); not including land or animal ownership (inclusion of these variables in the model, either together or separately, did not strengthen the index but rather weakened it); including the number of persons per room as a 3-level categorical variable and including the water and sanitation variables as 3-level categorical variables.

Step 2: Use MCA to assign loading to each variable and calculate asset index score.

MCA was applied to the data set using Stata **mca** command.

mca radio tv cellphone watch generator bike motorcycle electricity cookcharcoal latrinecat watercat floorcement wallcement roofmetal personroom3

The first dimension explained 76.4% of the variation. Factor scores for each variable included in the index are shown in **Table 1**.

Overall index scores were calculated (**predict** rowscores) for each household. As expected, this variable had a mean of 0 and standard deviation of 1. Figure 1 presents a histogram of these results.

Step 3: Divide households into wealth index categories, based on scores.

All individuals usually present in each household were assigned the household's standardized wealth index score, and all individuals in the sample population were ranked according to that score. The sample population was then divided into quintiles of individuals, with all individuals in a single household being assigned to the same quintile.

Step 4: Assess evidence for "internal coherence"

In order to determine whether the resulting index was performing according to expectations, several tests for internal coherence were conducted. The proportion for each asset variable was compared across the ranking levels, in order to determine if ownership of assets increased with wealth index ranking. See Table 2 for these results.

Limitations of the wealth index

The main limitation of the wealth index is how well it approximates current household wealth. One critique has suggested that this type of index can be interpreted not only as a measure of wealth but also of "involvement in the modern cash economy as opposed to the traditional sector" (Bingenheimer, 2007, p.83). Given the high positive weighting given to ownership of durable assets and absence of more traditional forms of wealth such as livestock or agricultural land, there is a tendency for the index to rate rural farming households as less wealthy.

The wealth index was equivalized (adjusted for household size); however, most of the variables included are shared by all household members and so this may or may not be optimal (Rutstein and Johnson, 2004).

The set of core assets for which data was collected in these surveys was based on standard survey protocol. No exploration was made of what specific indicators would best predict socio-economic position in this specific context. Therefore, the degree to which these assets are predictive of wealth may vary between settings and over time (Howe et al., 2008).

Following Table 1 are the results for each of the three rounds of data, November 2011 (**Table 2**), January 2012 (**Table 3**) and May 2012 (**Table 4**).

Table 1: Variables collected in the surveys, with results presented from November 2011

Asset variable		N	Mean (SD)
**Ownership of household goods**			
Has radio		720	50.46
Has television		121	8.48
Has refrigerator		6	0.42
Has cellphone		570	39.94
Has landline phone		19	1.33
Has stove		6	0.42
Has fan		43	3.01
Has watch		568	39.80
Has generator		54	3.78
Has gas lamp		18	1.26
Has bicycle		176	12.33
Has motorcycle		218	15.28
Has car		7	0.49
Has tractor		3	0.21
**Dwelling unit construction & characteristics**			
No. of members per sleeping room , mean (SEM)		1427	2.70 (0.04)
Floor material	*Carreaux, granite*	4	0.28
	Moquette, gerflex	9	0.63
	Cement	876	61.39
	Earthen	537	37.63
	Wood	1	0.07
Wall material	*Motte de terre, banco*	1116	78.21
	Bamboo/wood	1	0.07
	Brick/pierre	88	6.17
	Parpaing de ciment	144	10.09
	Semi-dur, banco recouvert	78	5.47
Roof material	*Dalle*	17	1.19
	Corrugated metal	1178	82.55
	Tile	9	0.63
	Terre	11	0.77
	Straw	212	14.86
Cooking fuel source	*Gas*	6	0.42
	Charcoal	153	10.72
	Wood	1268	88.86
**Access to services and resources**			
Has electricity		160	11.21
Drinking water source	*Piped/tap water*	258	18.08
	Borehole/well	220	15.42
	Springwater	289	20.25
	Rainwater	3	0.21
	Charrette avec citerne	1	0.07
	Surface water (river)	655	45.90
	Mineral water (bottled/sachet)	1	0.07
Sanitation facilities	*Flush toilet*	39	2.73
	Latrine	698	48.91
	Suspended latrine/toilet	2	0.14
	Field/bush	636	44.57
	Other	52	3.64
Owns agricultural land		851	59.64
Owns animals		1068	74.84

Table 2: Frequencies and factor scores for variables included in wealth index in November 2011

Asset variable	Value	Nov 2011			Wealth quintile				
		N	%	Factor score	1-highest	2	3	4	5-lowest
					269 (18.9)	297 (20.8)	274 (19.2)	304 (21.3)	283 (19.8)
**Ownership of household goods**									
**Radio**	No	707	49.54	0.861					
	Yes	720	50.46	-0.846	78.07	65.99	51.82	33.55	24.73
**Television**	No	1306	91.52	0.454					
	Yes	121	8.48	-4.903	43.87	0.67	0.36	0	0
**Cellular phone**	No	857	60.06	1.070					
	Yes	570	39.94	-1.609	88.48	61.28	35.77	15.79	1.41
**Watch**	No	859	60.20	0.819					
	Yes	568	39.80	-1.238	71.75	56.90	39.05	21.05	12.37
**Generator**	No	1373	96.22	0.130					
	Yes	54	3.78	-3.312	15.99	3.03	0.36	0.33	0
**Bicycle**	No	1251	87.67	0.089					
	Yes	176	12.33	-0.635	18.59	14.81	12.04	10.53	6.01
**Motorcycle**	No	1209	84.72	0.404					
	Yes	218	15.28	-2.239	42.38	18.52	10.95	4.93	1.41
**Dwelling unit construction & characteristics**									
**Floor material**	dirt/wood	538	37.70	1.394					
	cement	889	62.30	-0.843	92.94	85.19	70.44	56.91	7.07
**Wall material**	Mud brick	1117	78.28	0.610					
	cement	310	21.72	-2.197	59.85	33.00	14.96	2.96	0.35
**Roof material**	straw	223	15.63	1.719					
	aluminium	1204	84.37	-0.318	97.77	94.95	91.24	85.20	53.00
**Cooking fuel source**	Wood	1268	88.86	0.316					
	charcoal/gas	159	11.14	-2.520	33.09	12.46	7.30	3.62	0.71
**No. of members per sleeping room**	0.5 to 1.5 members	328	22.99	-0.326	26.77	25.59	25.55	19.74	17.67
	1.6 to 4.0 members	917	64.26	-0.025	66.54	64.65	63.50	65.79	60.78
	>4.0 members	182	12.75	0.711	6.69	9.76	10.96	14.47	21.55
**Access to services and resources**									
**Has electricity**	No	1267	88.79	0.512					
	Yes	160	11.21	-4.052	53.16	4.71	1.09	0	0
**Drinking water source**	piped/tap water	259	18.15	-1.712	44.24	22.56	11.31	9.21	4.95
	borehole/protected well	184	12.89	-1.059	21.56	18.52	11.31	7.57	6.01
	surface water (river, canal, rain, springwater)	984	68.96	0.649	34.20	58.92	77.37	83.22	89.05
**Sanitation facilities**	flush toilet	39	2.73	-1.472	5.20	4.38	2.19	1.64	0.35
	covered/improved latrine	229	16.05	-2.082	44.98	17.85	13.50	4.28	1.77
	bush/field/open latrine/other	1159	81.22	0.461	49.81	77.78	84.31	94.08	97.88

Table 3: Frequencies and factor scores for variables included in wealth index in January 2012 (N=1468)

Asset variable	Value	Jan 2012 N	Jan 2012 %	Jan 2012 Factor score	Wealth quintile 1-highest 294 (20.0)	2 294 (20.0)	3 295 (20.1)	4 292 (19.9)	5-lowest 293 (20.0)
**Ownership of household goods**									
**Radio**	No	681	46.4	0.912					
	Yes	787	53.6	-0.789	76.9	72.1	52.5	38.0	28.3
**Television**	No	1323	90.1	0.446					
	Yes	145	9.9	-4.071	41.5	6.5	1.0	0.3	0
**Cellular phone**	No	827	56.3	1.064					
	Yes	641	43.7	-1.373	83.7	63.6	39.3	23.0	8.5
**Watch**	No	881	60.0	0.873					
	Yes	587	40.0	-1.310	73.8	57.5	38.3	18.8	11.3
**Generator**	No	1397	95.2	0.168					
	Yes	71	4.8	-3.308	20.4	2.7	1.0	0	0
**Bicycle**	No	1315	89.6	0.125					
	Yes	153	10.4	-1.071	16.7	16.0	12.2	4.1	3.1
**Motorcycle**	No	1268	86.4	0.323					
	Yes	200	13.6	-2.046	34.7	17.7	9.8	4.1	1.7
**Dwelling unit construction & characteristics**									
**Floor material**	dirt/wood	513	35.0	1.650					
	cement	955	65.0	-0.886	96.9	88.4	73.6	58.9	7.2
**Wall material**	Mud brick	1140	77.7	0.553					
	cement	328	22.3	-1.923	54.4	29.9	21.0	4.8	1.4
**Roof material**	straw	273	18.6	1.918					
	aluminium	1195	81.4	-0.438	98.6	96.6	88.5	79.5	43.7
**Cooking fuel source**	Wood	1351	92.0	0.294					
	charcoal/gas	117	8.0	-3.391	29.3	6.8	2.7	0.7	0.3
**No. of members per sleeping room**	0.1 to 1.5 members	361	24.6	-0.253	28.9	26.9	27.5	20.9	18.8
	1.6 to 4.0 members	951	64.8	-0.028	66.0	62.2	64.8	70.2	60.8
	>4.0 members	156	10.6	0.755	5.1	10.9	7.8	8.9	20.5
**Access to services and resources**									
**Has electricity**	No	1310	89.2	0.465					
	Yes	158	10.8	-3.854	45.6	6.5	1.0	0.7	0
**Drinking water source**	piped/tap water	188	12.8	-2.457	38.1	12.2	9.5	2.7	1.4
	borehole/protected well	269	18.3	-1.262	33.3	32.3	11.2	11.3	3.4
	surface water(river,canal,rain spring)	1011	68.9	0.793	28.6	55.4	79.3	86.0	95.2
**Sanitation facilities**	flush toilet	17	1.2	-3.230	4.8	0.7	0.3	0	0
	covered/improved latrine	316	21.5	-1.461	42.5	28.2	20.7	10.3	5.8
	open latrine[§]	422	28.8	0.155	22.8	32.7	28.1	31.2	29.0
	bush/field/other	713	48.6	0.633	29.9	38.4	50.9	58.6	65.2

Table 4: Frequencies and factor scores for variables included in wealth index in June 2012 (N=1555)

Asset variable	Value	May 2012			Wealth quintile				
		N	%	Factor score	1-highest	2	3	4	5-lowest
					311 (20.0)	311 (20.0)	312 (20.1)	311 (20.0)	310 (19.9)
**Ownership of household goods**									
**Radio**	No	758	48.75	1.022					
	Yes	797	51.25	-0.972	81.99	72.99	54.81	31.51	14.84
**Television**	No	1387	89.20	0.561					
	Yes	168	10.80	-4.633	53.05	0.96	0.00	0.00	0.00
**Cellular phone**	No	846	54.41	1.127					
	Yes	709	45.59	-1.345	89.39	69.77	42.31	19.94	6.45
**Watch**	No	902	58.01	0.859					
	Yes	653	41.99	-1.187	77.81	57.88	41.67	23.47	9.03
**Generator**	No	1473	94.73	0.193					
	Yes	82	5.27	-3.464	21.86	2.89	1.28	0.32	0.00
**Bicycle**	No	1388	89.26	0.147					
	Yes	167	10.74	-1.225	21.22	15.43	8.97	5.79	2.26
**Motorcycle**	No	1313	84.44	0.439					
	Yes	242	15.56	-2.280	44.05	19.29	8.97	4.82	0.65
**Dwelling unit construction & characteristics**									
**Floor material**	dirt/wood	469	30.16	1.501					
	cement	1086	69.84	-0.648	95.82	87.78	77.56	63.99	23.87
**Wall material**	Mud brick	1127	72.48	0.563					
	cement	428	27.52	-1.481	58.84	36.66	22.44	17.68	1.94
**Roof material**	straw	220	14.15	1.717					
	aluminium	1335	85.85	-0.283	98.71	95.50	89.42	83.60	61.94
**Cooking fuel source**	Wood	1460	93.89	0.269					
	charcoal/gas	95	6.11	-4.138	26.37	3.22	0.32	0.64	0.00
**No. of members per sleeping room**	0.1 to 1.5 members	379	24.37	-0.122	25.72	25.72	24.04	25.08	21.29
	1.6 to 4.0 members	1000	64.31	-0.129	67.85	68.81	64.42	63.34	57.10
	>4.0 members	176	11.32	0.994	6.43	5.47	11.54	11.58	21.61
**Access to services and resources**									
**Has electricity**	No	1387	89.20	0.459					
	Yes	168	10.80	-3.787	43.73	8.36	1.28	0.64	0.00
**Drinking water source**	piped/tap water	148	9.52	-1.863	21.86	9.32	8.97	5.47	1.94
	borehole/protected well	385	24.76	-0.848	41.16	33.12	26.92	14.79	7.74
	surface water(river,canal,rain spring)	1022	65.72	0.589	36.98	57.56	64.10	79.94	90.32
**Sanitation facilities**	flush toilet	19	1.22	-5.692	5.79	0.32	0.00	0.00	0.00
	covered/improved latrine	269	17.30	-1.517	38.91	21.86	15.06	9,32	1.29
	open latrine§	490	31.51	0.116	26.37	34.08	33.97	39.87	23.23
	bush/field/other	777	49.97	0.591	28.94	43.73	50.96	50.80	75.48

Results for the analysis of access and ITN use by household wealth, as determined by quintile membership. A socioeconomic score was allocated to each household using a multiple correspondence analysis of household assets. Households were divided into five equal groups (quintiles) according to their level of wealth, and by definition approximately 20% were in each quintile. Quintile 1 represented the richest households and quintile 5 the poorest households. Each access and use indicator was assessed by socioeconomic quintile using PROC surveyfreq and PROC surveylogistic in SAS to determine if any indicators varied significantly as a function of wealth. P values are for test of differences (using Rao-Scott chi-square goodness-of-fit tests) in indicator among all five wealth quartiles (overall) and tests of contrast between poorest quartiles (Q4&5) vs richest quartiles (Q1&2) when the overall test was significant.

	**% individuals with access to at least 0.5 ITN**	**% individuals who slept under an ITN the previous night**	**% children under five who slept under an ITN the previous night**
**November**			
**P-value: overall**	0.04	0.01	0.79
**P-value: Q4&5 vs Q1&2**	83.6% vs 86.4% (p=0.04)	74.4% vs 67.6% (p=0.003)	
**January**			
**P-value: overall**	0.14	0.40	0.45
**P-value: Q4&5 vs Q1&2**			
**June**			
**P-value: overall**	0.39	0.37	0.79
**P-value: Q4&5 vs Q1&2**			

References

Bingenheimer, J. B. (2007). Wealth, wealth indices and HIV risk in East Africa. *Int Fam Plan Perspect* 33(2): 83-4.

Booysen, F., S. Van Der Berg, et al. (2008). Using an asset index to assess trends in poverty in seven sub-Saharan African countries. *World Development* 36(6): 1113-1130.

Filmer, D. and L. H. Pritchett (2001). Estimating wealth effects without expenditure data--or tears: an application to educational enrollments in states of India. *Demography* 38(1): 115-32.

Houweling, T. A., A. E. Kunst, et al. (2003). Measuring health inequality among children in developing countries: does the choice of the indicator of economic status matter? *Int J Equity Health* 2(1): 8.

Howe, L. D., J. R. Hargreaves, et al. (2008). Issues in the construction of wealth indices for the measurement of socio-economic position in low-income countries. *Emerg Themes Epidemiol* 5: 3.

Rutstein, S. O. and K. Johnson (2004). The DHS Wealth Index. Calverton, Maryland, ORC Macro.

Vyas, S. and L. Kumaranayake (2006). Constructing socio-economic status indices: how to use principal components analysis. *Health Policy Plan* 21(6): 459-68.
