# Supplementary material for: Effectiveness of post-campaign, door-to-door, hang-up, and communication interventions to increase long-lasting, insecticidal bed net utilization in Togo (2011–2012): a cluster randomized, control trial
Source: Malar J. 2014 Jul 9;13:260. doi: 10.1186/1475-2875-13-260 (PMC4110632; doi:10.1186/1475-2875-13-260)
Supplement: Additional file 2 — Hang-up and door-to-door visit monitoring results from the study area cantons as reported by volunteers. Description of data: Monitoring data collected by Togo Red Cross volunteers and staff during the implementation of the three phases of the hang-up interventions. Data provided by Karen Bramhill, the IFRC Operations Research Delegate overseeing the intervention implementation, and Ben Adinoyi, the Africa Health and Care Coordinator at IFRC. [file 1475-2875-13-260-S2.pdf]

Additional file 2: Hang-up and Door-to-Door visit monitoring results from the study area cantons as reported by volunteers

**Indicator**	HU	First DTD	Second DTD
Number of communities (cantons)	212 (20)	101 (10)	54 (5)
Number of households visited (% of total)	34,758 (89.4)	16,573 (88.7)	8094 (90.3)
% of households that received key messages	99.5	99.2	100.0
% of nets already hanging in household	57.5	91.9	96.0
% of nets hung by volunteer	33.6	1.3	0.8
% of nets hanging following the HU visit	91.2	93.3	96.8
