# Supplementary material for: Effectiveness of post-campaign, door-to-door, hang-up, and communication interventions to increase long-lasting, insecticidal bed net utilization in Togo (2011–2012): a cluster randomized, control trial
Source: Malar J. 2014 Jul 9;13:260. doi: 10.1186/1475-2875-13-260 (PMC4110632; doi:10.1186/1475-2875-13-260)
Supplement: Additional file 3 — Reasons for non-use by household access. Description of data: Reasons cited why a person did not use a net the previous night by study arm (unweighted) for households with sufficient access to ITNs (at least one ITN per two people) and without for the second and third evaluations (January and June 2012). [file 1475-2875-13-260-S3.pdf]

Additional file 3: Reasons for non-use by household access.

Reasons cited for why a person did not use a net the previous night by study arm (unweighted) for households with sufficient access to ITNs (at least 1 ITN per 2 people) and without for the second and third evaluations (January and June 2012).

Reason given by respondent	Survey	Insufficient access Study arm					Sufficient access Study arm				
		Control	HU	HU + 1 DTD	HU + 2 DTD	Total	Control	HU	HU + 1 DTD	HU + 2 DTD	Total
N	Jan	86	129	103		232	196	105	103		208
	June	153	182	66	54	455	168	183	64	56	471
Not enough nets - %	Jan	52.3	52.7	64.1		56.3	5.6	21	20.4		13.4
	June	53.6	52.7	45.5	66.7	53.6	19	21.3	20.3	17.9	20
Too hot - %	Jan	5.8	7.8	9.7		7.9	13.3	9.5	14.6		12.6
	June	2.6	4.4	4.5	7.4	4.2	8.3	9.8	29.7	17.9	13
No mosquitoes - %	Jan	4.7	3.9	0		2.8	11.7	15.2	7.8		11.6
	June	3.9	4.4	10.6	1.9	4.8	4.8	2.2	0	0	2.5
Negative experience - %	Jan	4.7	5.4	12.6		7.5	9.7	6.7	16.5		10.6
	June	2	2.2	3	3.7	2.4	6.5	3.8	9.4	3.6	5.5
Other - %	Jan	32.6	30.2	13.6		25.5	58.7	46.7	37.9		50.2
	June	35.3	35.7	36.4	16.7	33.4	56	61.2	32.8	53.6	54.6
Don't know - %	Jan	0	0	0		0	1	1	2.9		1.5
	June	2.6	0.5	0	3.7	1.5	5.4	1.6	7.8	7.1	4.5
